# Case-finding of dementia in general practice and effects of subsequent collaborative care; design of a cluster RCT

**DOI:** 10.1186/1471-2458-12-609

**Published:** 2012-08-04

**Authors:** Pim van den Dungen, Eric P Moll van Charante, Harm W J van Marwijk, Henriëtte E van der Horst, Peter M van de Ven, Hein P J van Hout

**Affiliations:** 1 Department of General Practice and Elderly Care Medicine, EMGO Institute for Health and Care Research, VU University Medical Center Amsterdam, Van der Boechorststraat 7, 1081 BT Amsterdam, The Netherlands; 2 Department of General Practice, Academic Medical Center, University of Amsterdam, Meibergdreef 15, 1105 AZ Amsterdam, The Netherlands; 3 Department of Epidemiology and Biostatistics, VU University Medical Center Amsterdam, PO Box 7057, 1007 MB Amsterdam, The Netherlands

## Abstract

**Background:**

In the primary care setting, dementia is often diagnosed relatively late in the disease process. Case finding and proactive collaborative care may have beneficial effects on both patient and informal caregiver by clarifying the cause of cognitive decline and changed behaviour and by enabling support, care planning and access to services.

We aim to improve the recognition and diagnosis of individuals with dementia in general practice. In addition to this diagnostic aim, the effects of case finding and subsequent care on the mental health of individuals with dementia and the mental health of their informal carers are explored.

**Methods and design:**

Design: cluster randomised controlled trial with process evaluation.

Participants: 162 individuals ≥ 65 years, in 15 primary care practices, in whom GPs suspect cognitive impairment, but without a dementia diagnosis.

Intervention; case finding and collaborative care: 2 trained practice nurses (PNs) invite all patients with suspected cognitive impairment for a brief functional and cognitive screening. If the cognitive tests are supportive of cognitive impairment, individuals are referred to their GP for further evaluation. If dementia is diagnosed, a comprehensive geriatric assessment takes place to identify other relevant geriatric problems that need to be addressed. Furthermore, the team of GP and PN provide information and support.

Control: GPs provide care and diagnosis as usual.

Main study parameters: after 12 months both groups are compared on: 1) incident dementia (and MCI) diagnoses and 2) patient and caregiver quality of life (QoL-AD; EQ5D) and mental health (MH5; GHQ 12) and caregiver competence to care (SSCQ). The process evaluation concerns facilitating and impeding factors to the implementation of this intervention. These factors are assessed on the care provider level, the care recipient level and on the organisational level.

**Discussion:**

This study will provide insight into the diagnostic yield and the clinical effects of case finding and collaborative care for individuals with suspected cognitive impairment, compared to usual care. A process evaluation will give insight into the feasibility of this intervention.

The first results are expected in the course of 2013.

**Trial registration:**

NTR3389

## Background

General Practitioners (GPs) increasingly recognize the importance and benefits of a timely and explicitly disclosed dementia diagnosis [1]. Still, although individuals with cognitive impairments contact their physicians more frequently than patients without such impairment, dementia is often not recognised or diagnosed [2,3]. There are many barriers to diagnosis at both the physician and patient level. Barriers at the physician level include time constraints, insufficient knowledge and skills to diagnose dementia, therapeutic nihilism and fear to harm the patient. Nevertheless, the primary care setting provides unique opportunities for timely diagnosis of dementia.

Arriving at a more timely diagnosis and improving the quality of care for individuals with dementia in primary care is feasible. Perry et al. showed that educational interventions directed at both GPs and practice nurses resulted in substantial increase in adherence to *diagnostic* guidelines and in number of incident dementia diagnoses [4]. Downs et al. demonstrated that decision support software and training of GPs improved detection rates [5]. Vickrey et al. showed that collaboration of GPs with care managers led to substantial improvement in adherence to dementia *care* guidelines [6]. They also described positive effects on patient health-related quality of life and on caregiving quality. Until now, there is a scarcity of literature on the effects of the combination of case finding and subsequent collaborative care on the mental health of individuals with cognitive impairment and their informal caregivers. Moreover, the validity of dementia diagnoses by GPs in an earlier phase was not assessed in the abovementioned studies.

We aim to improve recognition of and care for individuals with dementia in general practice. We hypothesize that case finding, directed at individuals in whom GPs suspect cognitive impairment but without a diagnosis of dementia, has the potential to triple the number of incident dementia diagnoses. As diagnosis may be more difficult at an earlier stage, the validity of GPs’ incident diagnoses is assessed. Considering its heterogeneous prognosis, the use of the diagnostic label Mild Cognitive Impairment (MCI) by GPs is debatable. As it may become more relevant in the future we did include it in our outcomes [7]. In addition, preferences regarding cognitive testing and disclosure of dementia diagnoses are explored in individuals in whom cognitive decline is suspected but whose cognitive function was not yet assessed. Finally, effects of collaborative care on the mental health of individuals with dementia or MCI and on the mental health of their informal carergivers are assessed.

## Methods and design

### Design of the study

**Figure 1:**
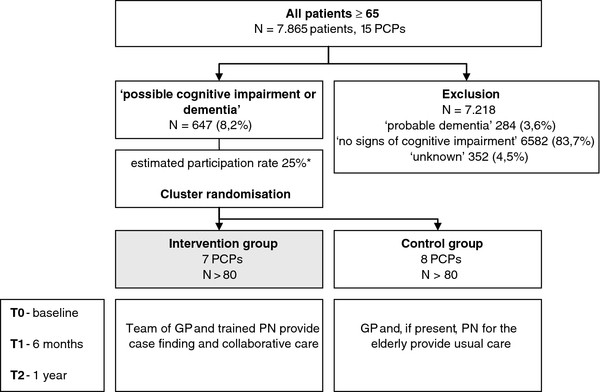
**Flow chart of the study design.** PCP = Primary Care Practice, GP = General Practitioner, PN = Practice Nurse. * based on a pilot study.

A cluster RCT is combined with a process evaluation. This design was chosen to prevent contamination between participants in both arms within practices. Before the training of GPs and practice nurses (PNs) and the deployment of the study PNs (seen intervention paragraph) Primary Care Practices (PCPs) are stratified based on the following potential effect modifiers: 1) practice nurse (PN) working specifically with elderly patients already present in PCP, 2) percentage of patients aged 65 years or older. Figure 1 provides a flow chart of the study design.

### Participants

#### Patients

GPs were presented with al list of the names and birth dates of *all* their patients aged 65 years or older and classified each individual as one of the following:

‘no signs of cognitive impairment’

‘possible cognitive impairment or dementia’

‘probable dementia’

‘unknown or no recent contact’

Individuals classified as having ‘possible cognitive impairment or dementia’ (according to their GP) and their informal caregivers, if present were eligible for the trial. GPs were asked to base their classification of cognition solely on their impression of individuals during previous contacts. GPs were allowed to use the medical records of patients, but not allowed to perform additional cognitive tests. Individuals all ready diagnosed with dementia were excluded.

The following exclusion criteria were used:

• Diagnosis of ‘probable dementia’ by GP or specialist;

• ‘No signs of cognitive impairment’ according to GP;

• Cognitive status ‘unknown’ to GP;

• Terminal illness patient or informal caregiver;

• Permanent admission to a nursing home expected within 6 months;

• Insufficient understanding of spoken Dutch or uncapable to express him- or herself.

### General practitioners and primary care practices

All 29 GPs working in the above described 15 PCPs participate in this study. GPs in the intervention group will collaborate with two practice nurses (PNs) specifically trained for this study; see intervention for more details.

### Setting

The study is executed in 15 PCPs in two towns near Amsterdam, The Netherlands, with 27.000 and 30.000 inhabitants respectively. In one town 4 duo-PCPs and 2 trio-PCPs participate in the study. In the second all GPs recently joined in one larger health centre. Within this centre they collaborate according to their original partnerships consisting of 6 duo-PCPs en 3 solo-PCPs.

### Interventions

The intervention in this study was designed by our project team comprising 3 GPs and 1 GP trainee, in close collaboration with two local GPs participating in the study. It is aimed at individuals classified as having ‘possible cognitive impairment or dementia’ by their GP and contains the following elements:

#### Training of GPs and practice nurses

In order to improve diagnosis and management of dementia in primary care, GPs and PNs undergo a training based on the effective training provided by Perry et al. in their study on case finding of dementia in primary care [8]. GPs will learn to recognize barriers to dementia diagnosis and learn how to diagnose dementia according to current guidelines (in particular the dementia guideline of the Dutch College of General Practitioners) [9]. Additionally, differential diagnosis and pharmacological treatment of behavioural problems will be addressed.

Practice nurses are trained to administer cognitive tests, to globally interpret the results and to present a conclusion on their cognitive and functional assessment to the GP. In addition, PNs are trained to administer the Resident Assessment Instrument – Home Care (RAI-HC), a standardised and extensively validated instrument for broad functional assessment of elderly patients and their informal caregiver [10]. They learn to make a care plan based on the RAI results and evaluate it periodically.

#### Case finding of MCI and dementia

**Figure 2:**
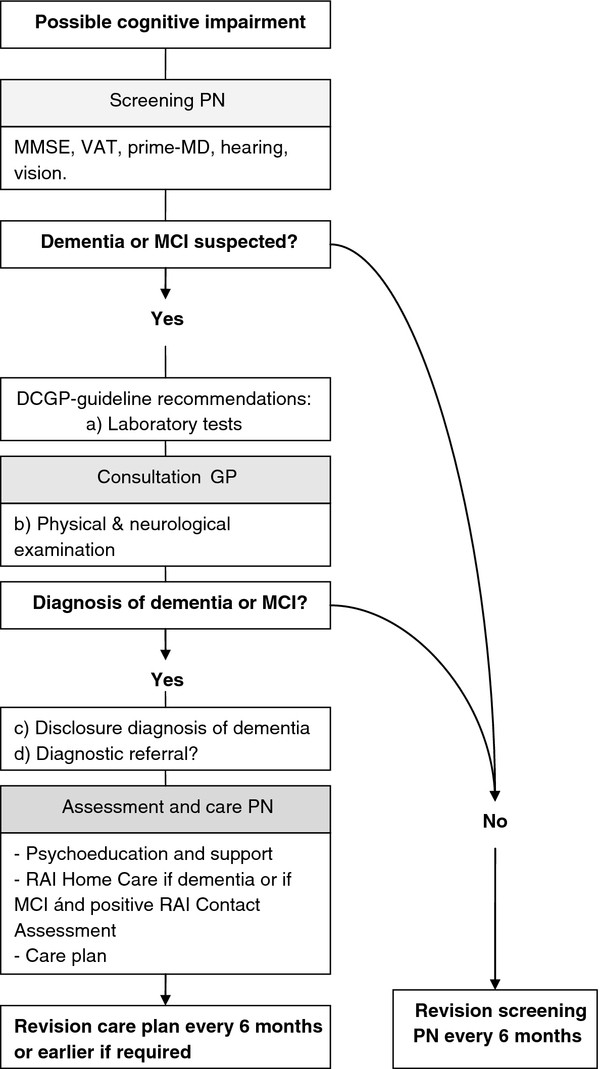
**Overview of the intervention.** PN = Practice Nurse, RAI = Resident Assessment Instrument, MMSE = Mini-Mental State Examination, VAT = Visual Association Test, Prime-MD = Primary Care Evaluation of Mental Disorders, DCGP = Dutch College of General Practitioners.

In the intervention practices two PNs are deployed who will perform several tests. They are exclusively involved with individuals with suspected cognitive impairment participating in the study. To all of them, they offer a brief screen of cognition (Mini Mental State Examination [MMSE] and Visual Association Test [VAT]), mood (Prime-MD), sensory functions (hearing and vision) and a brief assessment of need for home care by the RAI Contact Assessment (RAI-CA) [11]. Individuals with an MMSE score > 1 SD below the average MMSE of healthy individuals of comparable age and education and/or a VAT score ≤ 4 are referred to the GP for further evaluation [12,13]. GPs are trained, and supported by a brief practice guideline, to diagnose dementia according to the dementia guideline of the Dutch College of General Practitioners (DCGP). Thus, dementia diagnoses will be based on the criteria of the fourth edition of the Diagnostic and Statistical Manual of Mental Disorders (DSM-IV) and the diagnostic assessment includes blood tests [14]. To optimize diagnostic distinctiveness, GPs in the intervention practices are invited to also use the diagnostic category amnestic MCI, defined as results of the cognitive tests below the cut-offs with preserved social and occupational functioning [15]. In addition, the brief practice guideline provides an overview of drugs that may cause confusion or cognitive impairment in older individuals. Finally, the guideline provides criteria for diagnostic referral based on the dementia guideline of the DCGP. Figure 2 provides an overview of the intervention. 

#### Collaborative care

If dementia is diagnosed, or if MCI is diagnosed ánd the RAI-CA indicates an urgent need for care, the RAI-HC will be administered to assess further geriatric problems and needs. Based on the results of this assessment the PNs prioritise problems and prepare a care plan in consultation with the individual with MCI or dementia, the informal caregiver and the GP. In addition, the team of GP and PN provide information and support for the individual with cognitive impairment and, if present, for the informal caregiver.

Additionally, the PNs collect information on dementia services in the region and establish close collaboration with secondary care providers. The team of GP and PN will make agreements on collaboration with these providers based on National Collaboration Agreements for primary dementia care. These include for example agreements on information exchange, prescription of drugs, consultation and referral, crisis situations, (crisis-)admission.

The practice nurses serve patients of several PCPs. Patient contacts take place according to a predefined schedule. GPs remain responsible for all medical care, including crisis management during the study.

### Usual care

In the usual care group, GPs also explicitly classify the global cognitive functioning of their patients aged 65 and above, based on their recollection and, if needed, medical records.

Usual care for the group of patients consists of normal care as provided by GPs. GPs generally follow the guideline on dementia of the Dutch College of General Practitioners which adheres to the DSM-IV criteria for dementia diagnosis [9]. GPs in the usual care group may often lack a PN to assist in the diagnostic assessment of suspected cognitive impairment and in the provision of support and care coordination [16].

When dementia is suspected, GPs can refer the patient to a specialist or memory clinic for further diagnostic evaluation. Follow-up care may include referral to home care services or more specific services for dementia patients in the region. In general, no structured assessment of care needs or comprehensive care planning are performed. There are no explicit agreements on collaboration and referral among care providers in this region.

### Outcomes

The primary outcome is the number of incident MCI and dementia diagnoses after 12 months, in individuals suspected of cognitive problems by their GP.

This is operationalized as follows:

1. One year after the start of the intervention, GPs are asked to indicate whether they diagnosed:

• Mild cognitive impairment [15]

• Dementia syndrome [9]

• Cognitive impairment due to another cause, e.g.: mental retardation, cerebrovascular accident, traumatic brain injury, other brain disease.

• No cognitive impairment or diagnosis of dementia or MCI

2. Additionally, GPs are asked to indicate whether they explicitly disclosed the diagnosis of dementia to the individual and to his or her informal caregiver.

3. In parallel, the electronic medical records (EMR) will be checked, including medical correspondence, for dementia (and MCI) diagnoses by the GP and/or specialist. We chose to primarily ask GPs whether they have established a diagnosis, since documentation of the cognitive status in the EMR is limited and probably biased towards the intervention group [2].

Secondary outcome of the study will be the quality of life and mental health of individuals with dementia and their informal carers. Earlier identification of dementia and subsequent collaborative care may be beneficial but may also have negative effects on the secondary outcomes. We will explore these effects in the intervention and control group after 1 year. Furthermore, we will explore individuals’ preference regarding cognitive testing and disclosure of dementia diagnoses before and after the cognitive tests by the PN and, when indicated, further evaluation by the GP. Table 1 provides an overview of all measurements and their timing.

**Table 1:** Overview measurements

	**Instrument**	**Form**	**T0 – baseline**	**T1 – 6 months**	**T2 – 12 months**
**Individual with suspected cognitive impairment**					
GPs’ MCI or dementia diagnosis	1. GP asked to indicate dementia (and MCI) diagnoses of all study participants on a list				X
	2. Extraction dementia (and MCI) diagnoses from medical records				
Reference standard MCI or dementia diagnosis	CAMCOG [17]	Interview	X		X
Quality of life	QoL-AD [18] & EQ5D	Interview	X	X	X
Mood	MH5 (SF36) [19]	Interview	X		X
Preference regarding diagnostic evaluation of suspected cognitive impairment	Added questions	Interview and informed consent PN	X	X	X
**Informal caregiver**					
Quality of life	MDS & EQ5D	Questionnaire	X	X	X
Psychopathology	GHQ12 [20]	Interview	X		X
Sense of competence to provide care	SSCQ [21]	Interview	X	X	X
**Potential effect-modifiers / confounders**					
**Individual with suspected cognitive impairment**					
Sociodemography	By proxy	Interview	X		
Morbidity	By proxy	Interview	X		
Behavioural symptoms	NPI [22]	Interview	X		X
**Informal caregiver**					
Sociodemography	MDS	Interview	X		
Social support	SSL12 [23]	Questionnaire	X		X
Duration and intensity of caring	Added Q	Interview	X		
**GP / Primary Care Practice**					
Age GP	Added question	Questionnaire			
Sex GP	Added question	Questionnaire			
Presence of practice nurse for elderly patients	Added question	Questionnaire			
Attitude to diagnosis and care for individuals with dementia	Added questionnaire [24]	Questionnaire			
Percentage patients ≥ 65 years	-	Medical records			
Cluster size	-	Medical records			

*CAMCOG* = Cambridge Cognitive Examination, *MDS* = Minimal Dataset ZonMW, *QoL-AD* = Quality of Life in Alzheimer’s Disease, *EQ5D* = EuroQol utility questionnaire; 5 questions, *MH5 (SF36)* = 5 questions on Mental Health of the Short Form 36 questionnaire, *GHQ12* = General Health Questionnaire, *SSCQ* = Short Sense of Competence Questionnaire, *NPI* = Neuropsychiatric Inventory, *SSL12* = short version of the Social Support List.

Reference standard: validation of GPs’ incident dementia (and MCI) diagnosis.

In all participants, GPs’ dementia (and MCI) diagnoses will be compared to a reference standard diagnosis of cognitive status at baseline and at 1 year follow-up. The complete Cambridge Cognitive Examination (CAMCOG) and the memory section of the CAMCOG are used as reference standard to distinguish: 1) normal cognitive function (for age), from 2) amnestic MCI and 3) dementia [17]. We chose the CAMCOG because it is relatively brief and easy to administer and because of its good reliability and psychometric properties [25]. Previous studies showed a sensitivity and specificity in the differentiation of normal ageing from mild dementia of 93% and 87% respectively [25]. To optimize performance, Dutch normative data for age and education are used in the current study [26]. The sensitivity and specificity of the memory section for amnestic MCI were 78% and 74% respectively [17,27,28]. GPs and PNs are blinded to CAMCOG results of individual patients.

### Analysis and power calculation

#### Power calculation

Perry et al. studied the effect of education of GPs and collaboration with a practice nurse on incident dementia diagnoses in primary care. In their intervention group, dementia was diagnosed in 49.1% (130/265) and in their control group in 14.8% (20/135) of individuals suspected of cognitive impairment, after 1 year. In the study of Perry et al. GPs could only select 5 individuals in whom they suspected cognitive impairment, whereas in the current study, GPs are allowed to select all individuals in whom they suspect cognitive impairment. We asume that GPs will therefore include more patients of whom they are less certain that cognitive impairment is present, resulting in al lower prevalence of dementia. Therefore we estimate new dementia diagnoses to occur in 10% of individuals in the control group and 30% in the intervention group. The power analysis is based on a z-test for testing for a difference in proportions. Assuming a power of 70% and an alpha of 0.05 results in a required sample size of 49 individuals per arm. Assuming an average cluster size of 10 and an intra-class correlation between practices of 0.05 the design effect equals 1.45 and the required sample size corrected for clustering becomes 72 per arm. The planned sample size of 162 allows for 10% loss-to-follow up.

Based upon a pilot study, we anticipate a response rate of around 25% in the group individuals suspected of cognitive impairment. Therefore we decided to invite all 647 individuals classified as ‘possible cognitive impairment or dementia’ for study participation (see Figure 1).

### Statistical analysis

Difference in incident MCI and dementia diagnoses between intervention and control practices will be tested for using Generalized Estimating Equations (GEE) analysis. Clustering of the data within practices will be accounted for using an exchangeable correlation structure. An odds ratio for intervention and a 95% confidence interval will be computed as a measure for effect size. Baseline imbalances in principal characteristics are also tested for using GEE analysis. Potentially confounding or effect modifying variables are investigated by adding main effects and interactions with intervention to the model. Effect modification and confounding may occur at the physician level, e.g. the percentage of elderly patients in a practice, and at the patient level, e.g. the number of patients living alone.

Baseline imbalances in mental health between arms and effects of the intervention on mental health are also assessed using GEE analysis. Data will be analysed by the intention to treat principle.

### Randomisation

Practices are matched on percentage of patients aged 65 and above and on whether a practice nurse working specifically for older patients is present. R software is used to produce random numbers (1 or 2) assigning one of the matched practices to the intervention and the other to the control condition using a Bernouli distribution with probability 1/2. In some cases, GPs suspect cognitive impairment in both individuals of a pair. For these pairs random numbers (1 or 2) are drawn because we want to include only one individual per pair into the study.

### Bias handling

Several sources of bias potentially influence our outcomes. We will try to minimize bias and assess the presence and extent of the following sources of bias as follows:

1. Hawthorne effect and cognitive classification in the control group

In the control group, GPs are also asked to classify the cognitive function of all individuals aged 65 and over. This, in combination with taking part in the cluster RCT, will alert them to the presence of cognitive impairment in their patients and may increase the rate of MCI and dementia diagnoses. In addition, individuals in the control group are assessed with the CAMCOG. This may lead to higher awareness of cognitive problems and result in consultation of their GP with questions about their memory. This may reduce the contrast between intervention and control and may lead to an underestimation of the actual effect of the intervention.

2. Selection bias

Based on a small pilot study, we anticipate a relatively low response rate in the current study (25%). Although not previously described, we hypothesize the prevalence of cognitive impairment to be higher in the group of non-respondents. This would limit the external validity and feasibility of the study. We will therefore measure and report our main outcome, incident MCI and dementia diagnoses, in respondents and in non-respondents. To study selection bias, we will collaborate with GPs to collect anonymous data on the cognitive status of a selected sample of non-responders. In addition, sociodemographic data, data on some important risk factors for dementia and on factors associated with missed dementia diagnoses will be compared between responders and non-responders.

3. Index test and reference standard

To address time passed between index test (GP diagnosis) and reference standard (CAMCOG) and potential change of cognitive status in this period, the CAMCOG is administered before and after the index test; at baseline and at 1 year follow-up [2]. The index test will be regarded false positive if the reference standard is negative at baseline and at 1 year follow-up. The index test will be regarded false negative if the reference standard is positive at 1 year follow-up. Theoretically, the patients cognitive status may convert in this period between index test and reference standard, potentially resulting in a minimal overestimation of false negative cases and therefore a slight underestimation of the positive and negative predictive value and sensitivity of the index test.

4. Attrition bias

Patients with MCI or dementia may be more likely to prematurely end study participation [29,30]. This will mainly affect the measures of preference regarding diagnosis of cognitive impairment and measures of mental health. Reasons for discontinuation of participation will be collected and reported.

### Process evaluation

We will evaluate the process of implementation of the intervention and the feasibility of the intervention, both for care providers and for care receivers. Murray et al. describe factors to consider when assessing whether or not an intervention develops into routine care. Following this theoretical framework, we will consider *coherence* or meaning of the intervention to participants, *cognitive participation* or engagement of providers and study participants, whether *collective action* of providers occurs and *reflexive monitoring* or appraisal of the intervention by participants.

Information is used from meetings with GPs and PNs. In addition, a random sample of GPs is interviewed (semi-structured interviews) about their attitude towards dementia diagnosis and management and, for the intervention group, about how they evaluate the intervention. Moreover, we will interview a sample of individuals with cognitive impairment and their informal caregiver about their experiences with the intervention and the PN.

Quantitative data are collected and reported on the response rate, the number of patients eligible for the screen by the PNs and the number actually screened. In addition, information is collected on whether PNs report a clear and explicit conclusion to the GP after the screen and on whether individuals in whom the cognitive tests indicate cognitive impairment are actually evaluated by their GP. Finally, we assess whether individuals with an indication for collaborative care (Figure 2), do actually receive this care.

### Sub-study

In a sub-study we assess the prognostic value of GPs’ baseline classification of cognition of all their patients aged 65 and older (see participants paragraph). We compare their estimation of cognitive function to the CAMCOG at baseline to explore whether this cross-sectional classification procedure has potential as a first step in case finding of MCI and dementia in primary care.

### Ethics committee approval

Ethical approval for the study was obtained from the medical ethics committee of the VU University Medical Center Amsterdam, The Netherlands (reference number 2010/297). The study protocol is in accordance with the principles of the current version of the declaration of Helsinki. Written informed consent is obtained from all study participants.

## Discussion

Whether or not to discuss and explore signs of cognitive impairment, is a dilemma that GPs face regularly. In the current study half of the GPs is provided with extra knowledge to diagnose dementia, awareness of barriers to diagnosis, a practice guideline and a trained nurse to facilitate the diagnostic process. This will likely result in more, and ‘earlier’, diagnoses of MCI and dementia.

Studies of the effects of diagnosis on the mental health of recipients show conflicting results [31]. What has become clear is that relevant and conceivable information and support are very important in the period after diagnosis [31]. Therefore, we developed an intervention providing this follow-up support. Nevertheless, it is hard to predict the effects of case finding and collaborative care on the mental health of this group of patients with presumably relatively mild cognitive problems. We hope the current study will provide new insights into this complex domain. A potential limitation of the study is that the intervention is multifaceted. This prevents assessing how different aspects of the intervention, for example earlier diagnosis and support versus the comprehensive geriatric assessment, affect the mental health and quality of life of participants.

To our knowledge, this is the first study to explore patient preferences regarding diagnosis and disclosure of cognitive impairment in individuals in whom GPs suspect cognitive impairment but who were not yet referred for further cognitive assessment or diagnosed with dementia [31-35].

This study adds to the existing body of evidence by validating the incident GP diagnoses after 1 year of case finding. Previous studies showed the potential of GP and PN training for increasing the rate of dementia diagnoses, but there is still uncertainty about the true positive rate of earlier diagnoses. Indeed, increasing the number of incident dementia diagnoses may theoretically lead to more false positive diagnoses, with potential serious adverse effects on mental health and quality of life.

We aim to submit the first results of this study in the course of 2013.

## Abbreviations

CAMCOG: Cambridge Cognitive Examination; DCGP: Dutch College of General Practitioners; GEE analyses: Generalized Estimating Equations analyses; GP: General Practitioner; MCI: Mild Cognitive Impairment; MMSE: Mini-Mental State Examination; PCP: Primary Care Practice; PN: Practice Nurse; Prime-MD: Primary Care Evaluation of Mental Disorders; RAI: Resident Assessment Instrument; RCT: Randomised Controlled Trial; SD: Standard Deviation; VAT: Visual Association Test.

## Competing interests

The authors declare that they have no competing interests.

## Authors' contributions

PD and HH conceived the basic design and the main objectives of the study. PD drafted the manuscript and will coordinate data collection, statistical analysis and reporting of the results. HH, EM, HM, PV and HvdH participated in the design, helped to draft the manuscript and will support or participate in data collection, statistical analysis and reporting of the results. All authors read and approved the final manuscript.

## Pre-publication history

The pre-publication history for this paper can be accessed here:

http://www.biomedcentral.com/1471-2458/12/609/prepub

## References

[B1] Ahmad S Orrell M Iliffe S Gracie A GPs’ attitudes, awareness, and practice regarding early diagnosis of dementia Br J Gen Pract 2010 60 e360 e365 10.3399/bjgp10X515386 20849686 PMC2930246

[B2] van den Dungen P van Marwijk HW van der Horst HE Moll van Charante EP Macneil VJ van d V The accuracy of family physicians’ dementia diagnoses at different stages of dementia: a systematic review Int J Geriatr Psychiatry 2012 27 342 354 21626568 10.1002/gps.2726

[B3] Pentzek M Wollny A Wiese B Jessen F Haller F Maier W Apart from nihilism and stigma: what influences general practitioners’ accuracy in identifying incident dementia? Am J Geriatr Psychiatry 2009 17 965 975 10.1097/JGP.0b013e3181b2075e 20104054

[B4] Perry M Development and evaluation of a Dementia Training Programme for primary care 2011

[B5] Downs M Turner S Bryans M Wilcock J Keady J Levin E Effectiveness of educational interventions in improving detection and management of dementia in primary care: cluster randomised controlled study BMJ 2006 332 692 696 10.1136/bmj.332.7543.692 16565124 PMC1410839

[B6] Vickrey BG Mittman BS Connor KI Pearson ML la Penna RD Ganiats TG The effect of a disease management intervention on quality and outcomes of dementia care: a randomized, controlled trial Ann Intern Med 2006 145 713 726 17116916 10.7326/0003-4819-145-10-200611210-00004

[B7] Petersen RC Roberts RO Knopman DS Mild Cognitive Impairment. Ten Years later Arch Neurol 2009 66 12 1447 1455 Ref Type: Generic 10.1001/archneurol.2009.266 20008648 PMC3081688

[B8] Perry M Draskovic I van Achterberg T Borm GF van Eijken MIJ Lucassen P Can an EASYcare based dementia training programme improve diagnostic assessment and management of dementia by general practitioners and primary care nurses? The design of a randomised controlled trial BMC Health Serv Res 2008 8 71 10.1186/1472-6963-8-71 18384675 PMC2391160

[B9] Dementia Guideline of the Dutch College of General Practitioners (second revision); NHG-Standaard Dementie (Tweede herziening) http://nhg.artsennet.nl/kenniscentrum/k_richtlijnen/k_nhgstandaarden/NHGStandaard/M21_std.htm . 2003. Ref Type: Electronic Citation

[B10] Hawes C Fries BE James ML Guihan M Prospects and pitfalls: use of the RAI-HC assessment by the Department of Veterans Affairs for home care clients Gerontologist 2007 47 378 387 10.1093/geront/47.3.378 17565102

[B11] Hirdes JP Poss JW Curtin-Telegdi N The Method for Assigning Priority Levels (MAPLe): a new decision-support system for allocating home care resources BMC Med 2008 6 9 10.1186/1741-7015-6-9 18366782 PMC2330052

[B12] Lindeboom J Schmand B Tulner L Walstra G Jonker C Visual association test to detect early dementia of the Alzheimer type J Neurol Neurosurg Psychiatry 2002 73 126 133 10.1136/jnnp.73.2.126 12122168 PMC1737993

[B13] Kempen GI Brilman EI Ormel J [The Mini Mental Status Examination. Normative data and a comparison of a 12-item and 20-item version in a sample survey of community-based elderly] Tijdschr Gerontol Geriatr 1995 26 163 172 7570796

[B14] Reisberg B Diagnostic criteria in dementia: a comparison of current criteria, research challenges, and implications for DSM-V J Geriatr Psychiatry Neurol 2006 19 137 146 10.1177/0891988706291083 16880355

[B15] Petersen RC Negash S Mild cognitive impairment: an overview CNS Spectr 2008 13 45 53 18204414 10.1017/s1092852900016151

[B16] Bradford A Kunik M Schulz P Williams S Singh H Missed and Delayed Diagnosis of Dementia in Primary Care: Prevalence and Contributing Factors Alzheimer Dis Assoc Disord 2009 23 4 306 314 10.1097/WAD.0b013e3181a6bebc 19568149 PMC2787842

[B17] Roth M Tym E Mountjoy CQ Huppert FA Hendrie H Verma S CAMDEX. A standardised instrument for the diagnosis of mental disorder in the elderly with special reference to the early detection of dementia Br J Psychiatry 1986 149 698 709 10.1192/bjp.149.6.698 3790869

[B18] Thorgrimsen L Selwood A Spector A Royan L de Madariaga LM Woods RT Whose quality of life is it anyway? The validity and reliability of the Quality of Life-Alzheimer’s Disease (QoL-AD) scale Alzheimer Dis Assoc Disord 2003 17 201 208 10.1097/00002093-200310000-00002 14657783

[B19] Garratt AM Ruta DA Abdalla MI Buckingham JK Russell IT The SF36 health survey questionnaire: an outcome measure suitable for routine use within the NHS? BMJ 1993 306 1440 1444 10.1136/bmj.306.6890.1440 8518640 PMC1677883

[B20] Goldberg DP Blackwell B Psychiatric illness in general practice. A detailed study using a new method of case identification Br Med J 1970 1 439 443 5420206 10.1136/bmj.2.5707.439 PMC1700485

[B21] Vernooij-Dassen MJ Felling AJ Brummelkamp E Dauzenberg MG van den Bos GA Grol R Assessment of caregiver’s competence in dealing with the burden of caregiving for a dementia patient: a Short Sense of Competence Questionnaire (SSCQ) suitable for clinical practice J Am Geriatr Soc 1999 47 256 257 9988301 10.1111/j.1532-5415.1999.tb04588.x

[B22] Cummings JL Mega M Gray K Rosenberg-Thompson S Carusi DA Gornbein J The Neuropsychiatric Inventory: comprehensive assessment of psychopathology in dementia Neurology 1994 44 2308 2314 10.1212/WNL.44.12.2308 7991117

[B23] van Eijk LM Kempen GI van Sonderen FL A short scale for measuring social support in the elderly: the SSL12-I Tijdschr Gerontol Geriatr 1994 25 192 196 7974641

[B24] Kaduszkiewicz H van den BH Self-reported competence, attitude and approach of physicians towards patients with dementia in ambulatory care: results of a postal survey BMC Health Serv Res 2008 8 54 10.1186/1472-6963-8-54 18321394 PMC2289812

[B25] Huppert FA Brayne C Gill C Paykel ES Beardsall L CAMCOG–a concise neuropsychological test to assist dementia diagnosis: socio-demographic determinants in an elderly population sample Br J Clin Psychol 1995 34 Pt 4 529 541 8563660 10.1111/j.2044-8260.1995.tb01487.x

[B26] Jonker C Hooyer C The Amstel project: design and first findings. The course of mild cognitive impairment of the aged; a longitudinal 4-year study Psychiatr J Univ Ott 1990 15 207 211 2284372

[B27] Schmand B Walstra G Lindeboom J Teunisse S Jonker C Early detection of Alzheimer’s disease using the Cambridge Cognitive Examination (CAMCOG) Psychol Med 2000 30 619 627 10.1017/S0033291799002068 10883717

[B28] Gallagher D Mhaolain AN Coen R Walsh C Kilroy D Belinski K Detecting prodromal Alzheimer’s disease in mild cognitive impairment: utility of the CAMCOG and other neuropsychological predictors Int J Geriatr Psychiatry 2010 25 1280 1287 10.1002/gps.2480 21086538

[B29] Chatfield MD Brayne CE Matthews FE A systematic literature review of attrition between waves in longitudinal studies in the elderly shows a consistent pattern of dropout between differing studies J Clin Epidemiol 2005 58 13 19 10.1016/j.jclinepi.2004.05.006 15649666

[B30] Matthews FE Chatfield M Freeman C McCracken C Brayne C Attrition and bias in the MRC cognitive function and ageing study: an epidemiological investigation BMC Publ Health 2004 4 12 10.1186/1471-2458-4-12 PMC419705 15113437

[B31] Robinson L Gemski A Abley C Bond J Keady J Campbell S The transition to dementia–individual and family experiences of receiving a diagnosis: a review Int Psychogeriatr 2011 23 1026 1043 10.1017/S1041610210002437 21281553

[B32] Holroyd S Turnbull Q Wolf AM What are patients and their families told about the diagnosis of dementia? Results of a family survey Int J Geriatr Psychiatry 2002 17 218 221 10.1002/gps.552 11921148

[B33] Marzanski M Would you like to know what is wrong with you? On telling the truth to patients with dementia J Med Ethics 2000 26 108 113 10.1136/jme.26.2.108 10786321 PMC1733205

[B34] Jha A Tabet N Orrell M To tell or not to tell-comparison of older patients’ reaction to their diagnosis of dementia and depression Int J Geriatr Psychiatry 2001 16 879 885 10.1002/gps.412 11571768

[B35] Pinner G Bouman WP Attitudes of patients with mild dementia and their carers towards disclosure of the diagnosis Int Psychogeriatr 2003 15 279 288 10.1017/S1041610203009530 14756163

